# AM-251, A Cannabinoid Antagonist, Modifies the Dynamics of Sleep–Wake Cycles in Rats

**DOI:** 10.3389/fphar.2019.00831

**Published:** 2019-07-26

**Authors:** Emese Bogáthy, Noémi Papp, Szilvia Vas, György Bagdy, László Tóthfalusi

**Affiliations:** ^1^Department of Pharmacodynamics, Semmelweis University, Budapest, Hungary; ^2^MTA-SE, Neuropsychopharmacology and Neurochemistry Research Group, Budapest, Hungary; ^3^Department of Physiology, Development and Neuroscience, University of Cambridge, Cambridge, United Kingdom; ^4^NAP-A-SE, New Antidepressant Target Research Group, Budapest, Hungary; ^5^NAP-2-SE, New Antidepressant Target Research Group, Budapest, Hungary

**Keywords:** sleep–wake behavior, AM-251, sleep cycle, cannabinoid receptor, heatmap, REM sleep

## Abstract

**Study Objectives:** (a) To describe the microarchitecture of wakefulness and sleep following administrations of 5- and 10-mg/kg AM-251 in rats. (b) To develop a new statistical method to follow bout-to-bout dynamics.

**Method:** Wistar rats (n = 6) had been equipped with electroencephalography (EEG) and electromyography (EMG) electrodes. Following their recovery and habituation after the surgery, the animals were injected with vehicle and 5- and 10-mg/kg AM-251 intraperitoneally and EEG, EMG, and motor activity were analyzed for the subsequent 3 h.

**Results:** AM-251 induced a dose- and time-dependent increase in the number of bouts in active wake (AW), and it decreased this number in all other vigilance states except in passive wake (PW). In contrast, the bout duration in PW compensatory decreased. The effect of AM-251 on the sleep transition dynamics was monitored with a new tool we call “transition heatmap.” The analysis of bout trajectories with transition heatmaps reveals a highly organized pattern.

**Conclusion:** AM-251 selectively influences the frequency of vigilance state transitions, but it has no direct impact on the state lengths. AM-251 markedly changed the state transition dynamics, which was visualized with the help of state transition heatmaps.

## Introduction

Analyses of sleep–wake behavior have already confirmed that antagonizing CB1 and CB2 receptors disrupt the normal sleep pattern, although the results are contradictory. Suppression of rapid eye movement sleep (REMS) in rats has been reported with dronabinol, a nonselective CB1/CB2 antagonist (Calik and Carley, 2017), but also with the CB1 receptor–selective antagonist AM-251 (Santucci et al., 1996; Goonawardena et al., 2011). The REMS-reducing effect has been confirmed in mice too with the application of the CB1 receptor antagonists AM-251 and ABD459 (Goonawardena et al., 2015). However, regarding non-REM sleep (NREMS) states, Santucci et al. have found in rats that the amount of wakefulness is increased at the expense of slow-wave sleep (S), while Goonawardena et al. could not confirm this effect either in rats (Goonawardena et al., 2011) or in mice (Goonawardena et al., 2015). In line with this, neither the total amount nor the bouts of lengths of NREMS and wake episodes were affected by any receptor-selective and non-selective antagonists on the CB1 and CB2 receptors (Calik and Carley, 2017). In the latter studies, only three vigilance states (wakefulness, NREMS, and REMS) were distinguished (Santucci et al., 1996; Goonawardena et al., 2011; Goonawardena et al., 2015; Calik and Carley, 2017). Furthermore, both the number and bout duration data of vigilance states in treatment groups were compared with standard statistical tools, which have been developed for normally distributed data. However, it has been suggested recently that the application of more advanced statistical methods to model bout sequence allows a better insight into the underlying physiological mechanisms. Several methods have been proposed, such as survival analysis (Klerman et al., 2013), Markov chains (Bizzotto et al., 2010; Kostyalik et al., 2014), and state-space technique (Diniz Behn et al., 2010). It has also been observed that vigilance states, previously thought to be homogenous, can be further differentiated by the duration of bouts. Simasko et al. and McShane et al. have distinguished short and long wake bouts (Simasko and Mukherjee, 2009; McShane et al., 2010). Moreover, McShane et al. have argued that distributions of every standard vigilance state, such as REMS, NREMS, and wakefulness, can be decomposed into spikes (short but frequent bouts) and slabs (long-lasting but rare bouts). They have also showed that the bout characteristics depend on the previous states (McShane et al., 2013). Clearly, there is a sharp contradiction between the requirements of distinguishing more sleep states and using more sophisticated models. For example, a Markov chain model with six states such as active wake (AW), passive wake (PW), light-, and deep slow-wave sleep (S1 and S2, respectively), REMS and intermediate stage of sleep (IS) requires 36 parameters to be estimated if it is assumed that any state is achievable from any other. If additional time or drug effects are assumed, the number of parameters is multiplied further, so estimating all of them would be practically impossible. Typical solutions to resolve this conflict are i) restricting the number of states (like wake, REMS, and NREMS) or ii) setting some parameters arbitrarily to zero. Therefore, there is a clear need for analyses that are model-independent, yet still more informative than the commonly used statistics. McShane et al. (2013) suggested the application of three summary parameters for this purpose: i) the number of bouts—that is, conditional on the previous states; ii) the number of spikes (short bouts); and iii) the mean duration of long bouts. In this work, the applicability of the new explorative approach by McShane et al. (2013) is illustrated using our data. Additionally, prompted by the nature of the data itself, we propose new tools for explorative sleep state analysis.

## Methods

### Animal Maintenance

All animal experiments and housing conditions were carried out in accordance with the EU Directive 2010/63/EU and the National Institutes of Health “Principles of Laboratory Animal Care” (NIH Publications No. 85-23, revised 1985), as well as specific national laws (the Hungarian Governmental Regulations on animal studies 40/2013). The experiments were approved by the National Scientific Ethical Committee on Animal Experimentation. Male Wistar rats (n = 6) were provided by the Animal Facility (Semmelweis University, Budapest, Hungary). During the whole period of the experiment, the rats were kept under controlled environmental conditions (21 ± 1°C, 12 h/12 h light–dark cycle with light on at 10 am); food and water were available *ad libitum* during the whole experiment. On the day of the EEG surgery, the rats weighed 300–330 g. All efforts were made to minimize pain and discomfort of the rats.

### Surgery

The rats were chronically implanted with stainless steel screw electroencephalographic (EEG) electrodes epidurally. The surgery was performed under halothane (2%) anesthesia (Fluotec 3 vaporizer) using a Kopf stereotaxic instrument. For frontoparietal EEG recordings, the positions of the electrodes were: left frontal cortex (L = 2.0 mm and A = 2.0 mm to bregma) and left parietal cortex (L = 2.0 mm and A = 2.0 mm to lambda), as described earlier (Kantor et al., 2002). Also, a ground electrode was placed over the cerebellum. To detect muscular activity, stainless steel spring electromyographic (EMG) electrodes embedded in silicon rubber (Plastics One Inc., Roanoke, VA, USA) were implanted into the neck muscles on both sides. Following the implantation surgery, the rats were kept in a square glass recording chamber separately during the whole experiment.

### Drug Administration

AM-251 (AM, N-[Piperidin-1-yl]-5-[4-iodophenyl]-1-[2,4-dichlorophenyl]-4-methyl-1H-pyrazole-3-carboxamide) was purchased from Tocris Cookson (Bristol, UK). The vehicle of AM-251 was composed of the mixture of 70% PBS (phosphate buffered saline, pH = 7.4), 20% dimethyl sulfoxide, and 10% Tween 80. The rats were treated with an intraperitoneal (i.p.) injection of 5- or 10-mg/kg AM-251 or vehicle (Veh) at the beginning of passive phase (at 10 am). All rats were given all treatments in a cross-over design with 3 days washout.

### EEG Recording

After a 7-day recovery period, the rats were attached to the EEG system by a flexible recording cable and an electric swivel, fixed above the cages, permitting a free movement of the animals. The animals were attached to the EEG system a week before starting the cross-over of treatments and were kept connected to the system during the whole experiment. To detect the motor activity of the rats, electromagnetic transducers were used, in which potentials were generated by movements of the recording cable (Kantor et al., 2002). EEG, EMG, and motor activities were recorded during a 24-h-long period, starting at light onset. During the EEG recordings, the rats were undisturbed and had free access to standard rodent chow and tap water. The signals were amplified by analogue filters (Coulburn Lablinc System, USA; filtering below 0.50 Hz and above 100 Hz at 6 dB/octave) and subjected to analogue to digital conversion (MVRD-2200 V, Canopus, Japan) with a sampling rate of 256 Hz. Data were stored on a computer for further processing.

### Sleep Scoring

The vigilance states were analyzed using a semi-automatic method. First, we ran an automatic stage-scoring using SleepSign for Animal software for 4-s epochs (Kissei Comtec America, Inc., USA). It was followed by visual supervision. The following vigilance stages were classified based on a previous work of our laboratory (Kantor et al., 2002)—active wakefulness (AW): the EEG is desynchronized showing low-amplitude activity at beta (14–30 Hz) and alpha (8–13 Hz) frequencies accompanied by high EMG and intense motor activity; passive wakefulness (PW): the EEG is characterized by low-amplitude activity at beta (14–30 Hz) and alpha (8–13 Hz) frequencies accompanied by high EMG activity but minimal or no motor activity; light slow-wave sleep (S1): high-voltage slow cortical waves (0.5–4 Hz) interrupted by spindles (6–15 Hz) and accompanied by reduced EMG and no motor activity; deep slow-wave sleep (S2): with continuous high-amplitude slow cortical waves (0.5–4 Hz), minimal EMG, and no motor activity; REMS: desynchronized EEG activity with regular theta waves (5–9 Hz) were accompanied by silent EMG and motor activity with occasional muscular twitching; and IS: a brief stage just prior to or after REMS, characterized by an unusual association of high-amplitude spindle activity (mean 12.5 Hz) with low-frequency (mean 5.4 Hz) theta rhythm. The latency of NREMS was calculated as the time elapsed between the drug administration and the occurrence of the first consecutive NREMS (S1, S2, and IS) episode lasting at least 3 min and not interrupted by more than 14 consecutive 4-s epochs, or a cumulated total of 240 s not scored as NREMS (Huber et al., 1998).

### Statistical Analysis

This study is a three-period crossover design study with repeated measurements within the periods. A simple linear model was applied to assess the dependence of the response variables (bout number, bout durations, total sleep time) on the explanatory variables (treatment and time). The variable “treatment” was a categorical variable with three levels corresponding to three doses: 0, 5, and 10 mg/kg, respectively. The observation period was split into three intervals (1st h, 2nd h, and 3rd h), and the resulting time variable was included into the statistical model as an unordered categorical variable. Thus, the statistical model for bout numbers and bout durations had three independent variables: “treatment” and “time” as fixed factors and “animals” as a repeated measure variable.

Crossover design studies can be analyzed with repeated measures ANOVA or with mixed linear regression. The regression approach is more versatile and can be used even for not normally distributed response variables. Because of that, we chose the second option. The bout frequency was assumed following overdispersed Poisson distribution, and a mixed regression model variant called “negative binomial mixed regression” was used to handle overdispersion. Bout durations and total times were logarithmically transformed before fitting a regression model since their distributions were heavily right skewed.

Regression models were fitted with and without the variable “treatment” to evaluate the significance of the overall treatment effect. The assessment is based on F-test since the ratio of the corresponding residual mean squares follows F distribution. Using contrasts, the overall treatment effect was split into two parts corresponding to the differences between the effects of the 10-mg dose *versus* the Veh and of the 5-mg dose group *versus* the Veh. The difference estimates divided by their standard errors follow t-distribution, which allowed testing the treatment effects by simple t-tests at each dose level. We report the nominal p values without multiplicity adjustments because the applied two-step testing strategy controls the overall type I error. The difference estimates following fitting negative binomial regression models were raised to power because, in this form, they have a clear meaning: they show the bout numbers relative to the control. For practical significance assessment, we plotted them with the corresponding 95% confidence intervals. For bout duration, the parameter of interest was the time × treatment interaction, but the meaningful interpretation of the regression parameters was not straightforward. Therefore, we computed the 95% confidence intervals of the model predicted means to visualize the difference between the treatment groups. Lack of overlap between confidence intervals was taken as evidence of significant differences between treatments in the three time intervals. The linear model for “total time” had only one exploratory variable (treatment). In this special case, our testing approach is equivalent to what is known otherwise as one-way repeated measures ANOVA with random effects.

To study the conditional dependence of vigilance states, first, we cross-tabulated the number of bouts by the type of predecessor bouts. Tables obtained in this way were perceived as contingence tables between two nominal variables. Instead of the overall independence hypothesis (transitioning from any state to any other state is equally likely), we focused on the question of how likely it is that “state j” follows “state i.” This probability was estimated by calculating the standardized difference between the observed and expected probabilities for each cell in the table. Here “expected” means “according to the independence null hypothesis.” The resulting z-statistics asymptotically follows normal distribution, and values larger than 2 and smaller than -2 are usually considered as signs of significant dependence. The z-statistics cannot properly be estimated if there are less than five observations and, in such cases, z was not calculated. The idea of visualizing dependence between categories with the help of the z-statistics is coming from (Friendly, 1999). We used the freely available R software for computation and visualization (R Core Team, 2018) with several additional libraries including ggplot2 (Wickham, 2016) and lme4 (Bates et al., 2015).

## Results

### AM-251 Increases the Total Time Spent in Wakefulness

**Figure 1:**
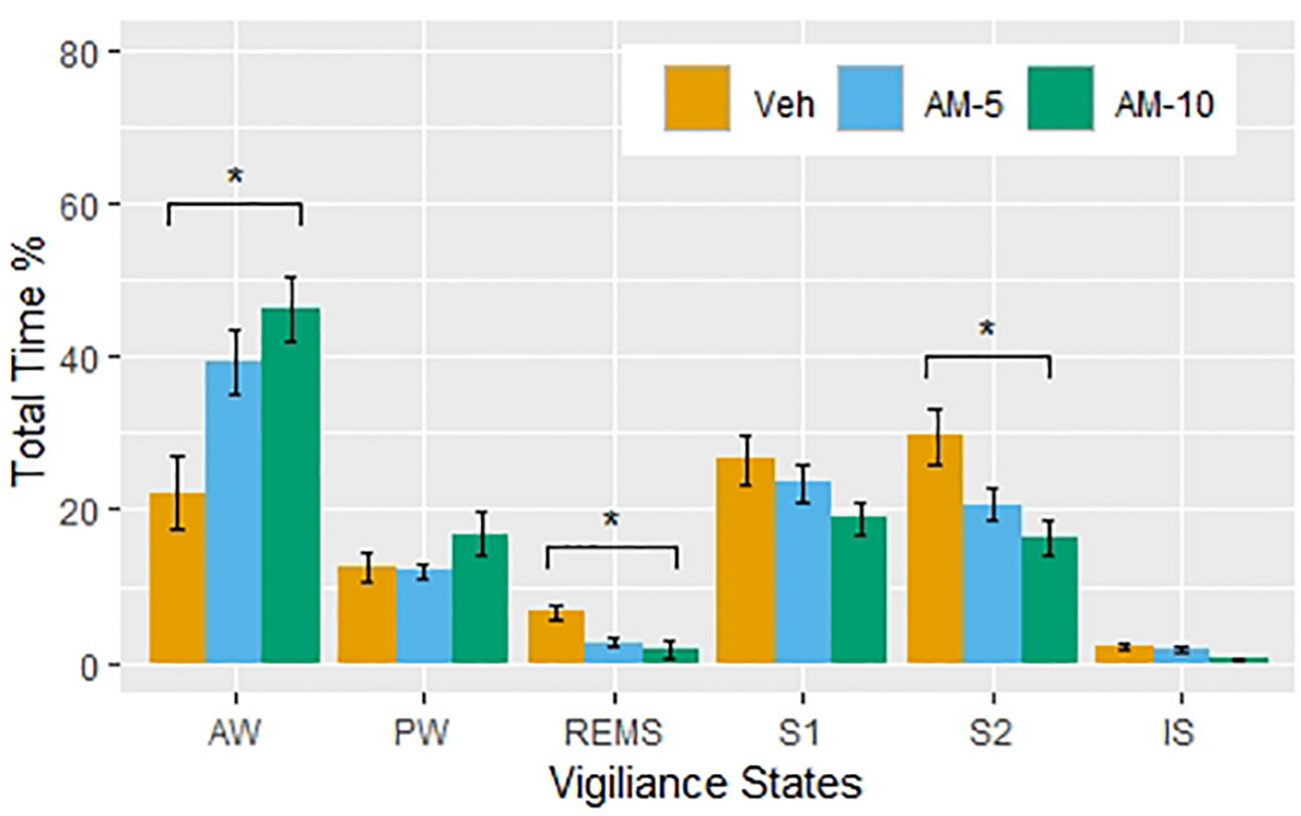
The percent of the time spent (means ± SEM, N = 6 per groups) in vigiliance states during the first 3 hours following intraperitioneal injections of vehicle (Veh) and AM-251 in 5-mg/kg (AM-5) and 10-mg/kg (AM-10) doses. Note that the larger dose significantly increased the time spent in active wake and decreased the time spent in rapid eye movement sleep and deep slow-wave sleep compared to the Veh group. *p < 0.05 compared to Veh.

The percentages of total time spent in vigilance states in the first 3 h after the treatment with the different doses of AM-251 is displayed in **Figure 1**. The amount of wakefulness is increased at the expense of REMS and NREMS stages. **Figure 1** shows a clear dose-dependence in the effect of AM-251, although a detailed statistical analysis showed that only the higher dose had significant effect in AW (t = 2.55, df = 50, p = 0.014), REM sleep (t = 2.19, DF = 28, p = 0.029), and S2 (t = 2.53, DF = 51, p = 0.0148). Despite the NREMS-suppressing effect of AM-251, the latency of NREMS did not change (repeated measures one-way ANOVA: F 2,10 = 0.4204, p = 0.6679).

### Shape Analysis of the Distribution of Bout Duration in Different Vigilance Stages

**Figure 2:**
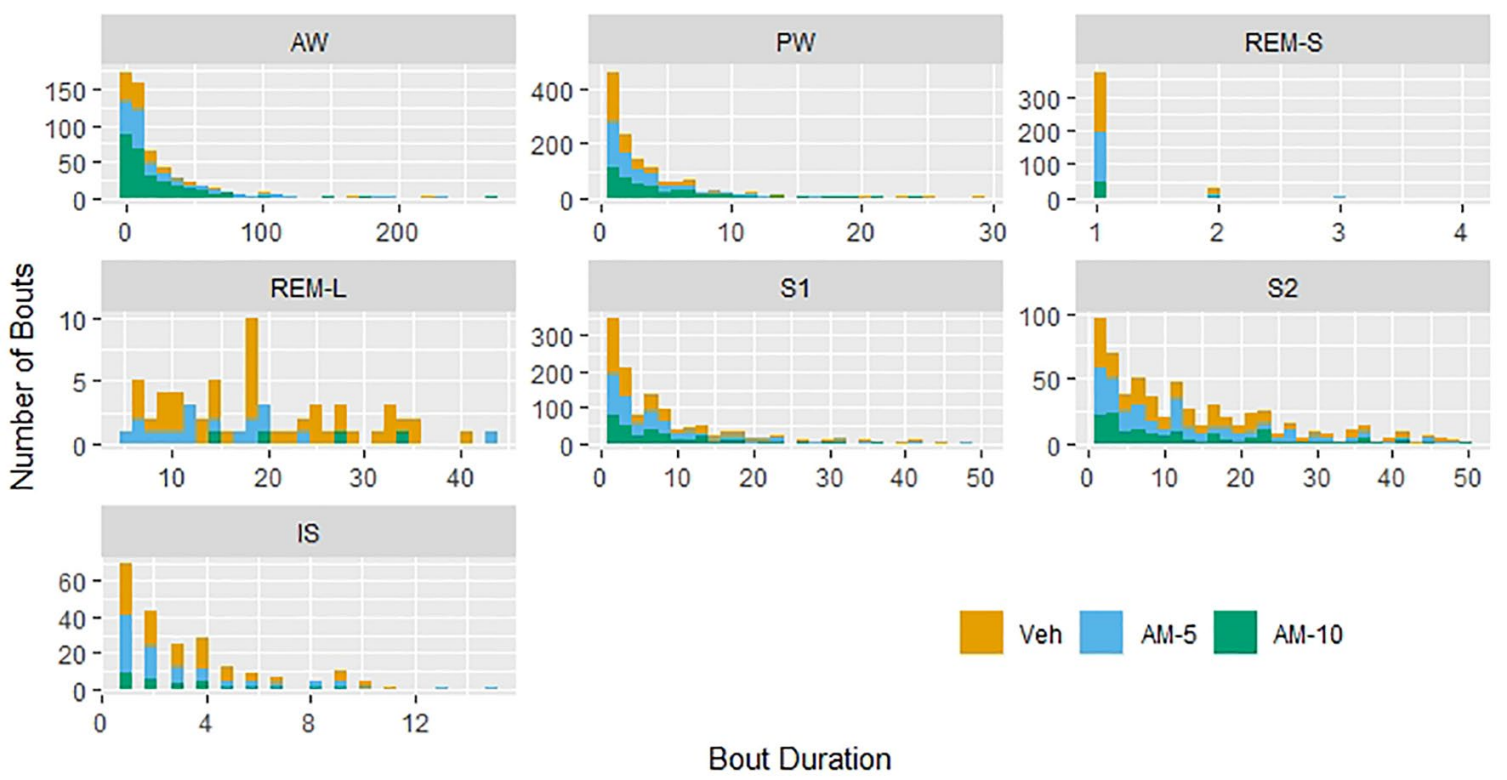
Histograms of bout durations of each vigilance stage. The Y axis shows the total counts of bouts in units of 4-s epochs during the 3-h-long observation period. The distributions of bout durations in rapid eye movement sleep (REMS) have two distinct components: a very short and relatively frequent bout, referred as “short REMS bout” (REMS-S) and the much longer and less frequent “long REMS bout” (REMS-L).

Histograms of bout durations in units of 4-s epochs are presented in **Figure 2**. As expected, the distributions are heavily right-skewed. Spikes (short, high frequency bouts) and slabs (long, low-frequency bouts) described by McShane et al. (2010) in mice can be observed in case of AW, PW, S1, and particularly in REMS. Therefore, we reclassified bouts of REMS as “short” (maximum length is 16 s, 4 epochs, REMS-S) and long REMS bouts (REMS-L). However, no major differences can be observed between the shapes of distributions in different treatment groups (**Figure 2**). If AM-251 had major effect on the bout durations, then histograms of treated groups would be shifted relative to the control. There is no sign of that in **Figure 2**.

### The Effect of AM-251 on Bout Frequencies

**Figure 3:**
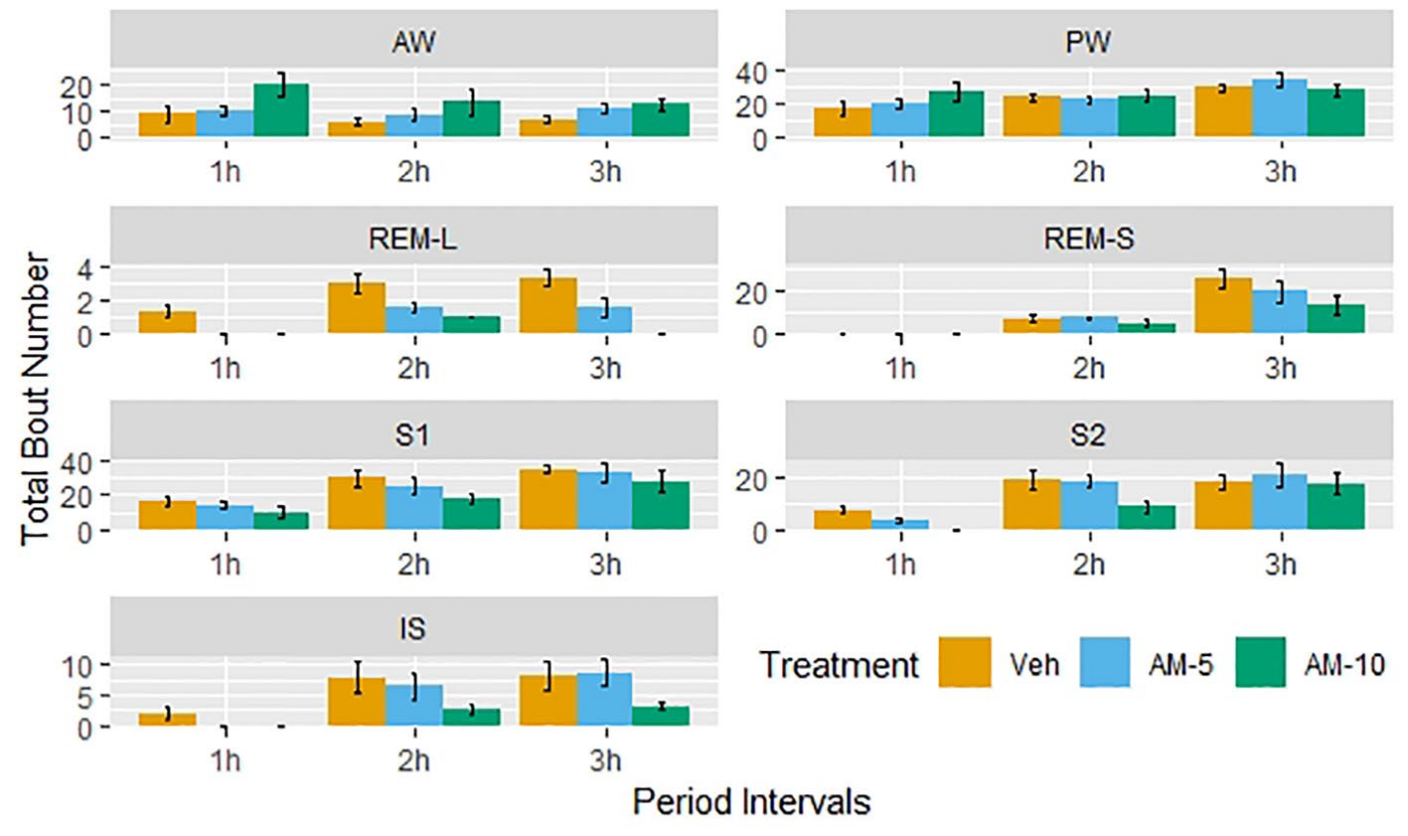
Dose-dependent effect of AM-251 on the number of bouts in different vigilance stages. Vehicle (Veh) or AM-251 was injected intraperitoneally at the beginning of passive phase in 5- and 10-mg/kg doses (AM-5 and AM-10). The 3-h length observation periods were divided into three 1-h intervals, and the bars show the number of episodes as means ± SEM (of N = 6 rats) by these intervals.

**Figure 4:**
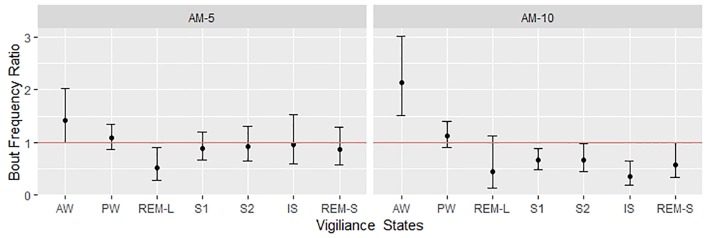
Bout frequency ratios were estimated with negative binomial regression. The figure shows the estimated ratios with the corresponding 95% confidence intervals. The effect is significant if the confidence interval crosses the red line (where the ratio is 1), which is corresponding to the “no effect” (also called null hypothesis).

The 3-h observation periods were divided into three segments, and the number of bouts by treatment groups is shown in **Figure 3**. A clear dose and time dependency can be observed additionally to circadian variation shown by the control group. Therefore, the time- and subject-adjusted bout frequency ratios are reasonable parameters to assess the drug effect. **Figure 4** gives the frequency ratios with the corresponding 95% confidence intervals. AM-251 applied in 5-mg/kg dose significantly suppressed the occurrence of REMS-L bouts and moderately increased the number of AW bouts. In the larger dose, AM-251 increased the number of AW bouts and decreased the number of bouts in all other vigilance states except PW. The effect on REMS-L is not significant, but it is still on the same level as with the lower dose (**Figure 3**).

### Effect of AM-251 on Bout Durations

Bout durations were analyzed by mixed linear regression following logarithmic transformation. The independent variables in the regression equation—time and treatment—were declared as categorical variables (i.e., factors). Significance of the variables was again evaluated by comparing residual mean squares. Each vigilance state was analyzed this way.

**Figure 5:**
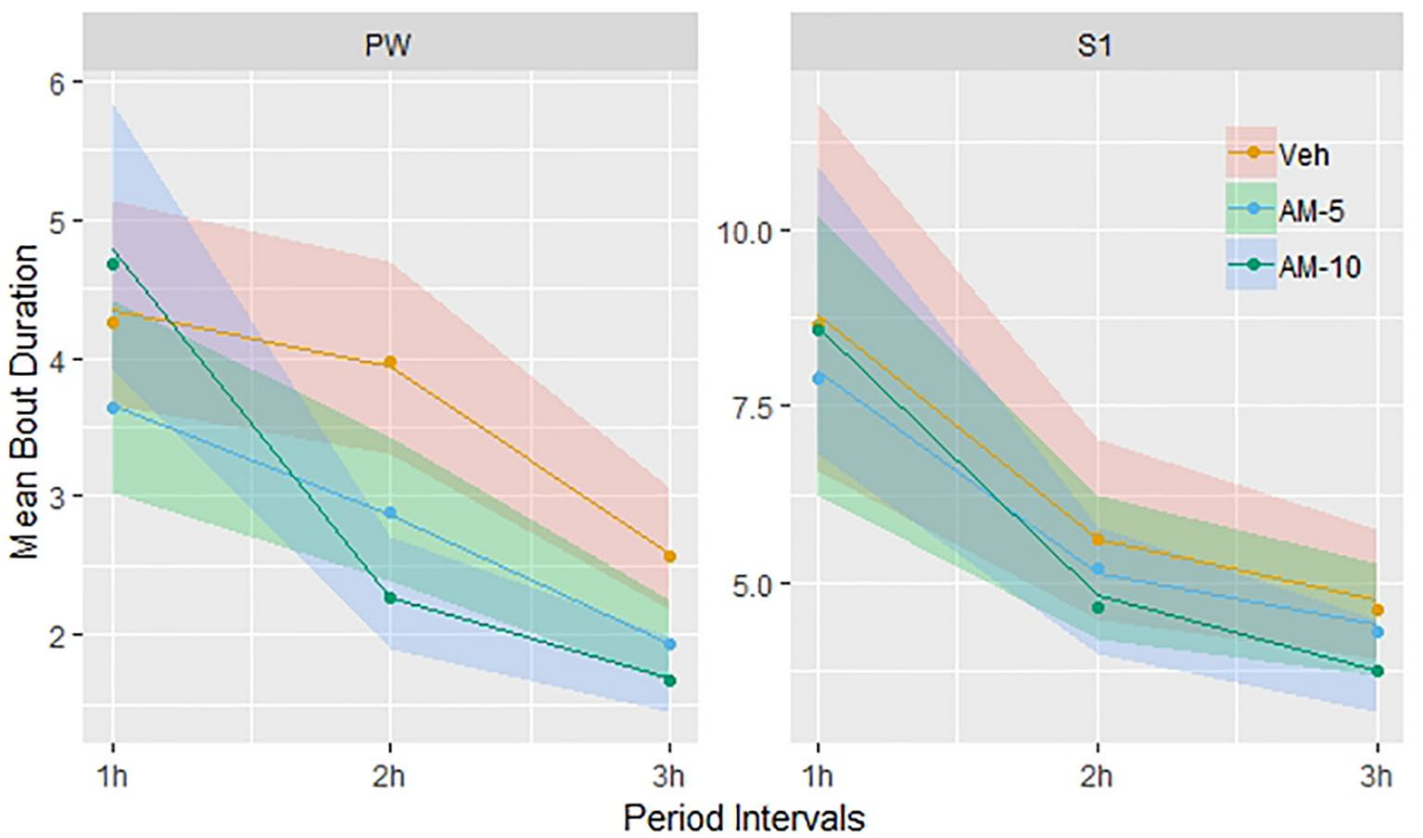
Dependence of bout durations on time and treatment was modeled using linear mixed regression. Before the analysis, bout durations were transformed logarithmically and the linear model included time and treatment and their interactions as categorical variables. The figure shows the observed means and the back-transformed confidence intervals of the fitted means (shown by colored stripes). The time trends are visually different but the time x treatment interaction is significant only for PW and only in the 10-mg/kg dose group.

The treatment effect of AM-251 was significant only in PW (F = 15.07, DF = 2, 1,352, p < 0.001) and moderate in S1 (**Figure 5**). In case of S1, only the 10-mg/kg dose of AM-251 had significant effect (t = 2.19, DF = 1,245, p = 0.0287). The interpretation of these statistical results is not straightforward; so, to get a better understanding, we plotted the observed means with the model predicted 95% confidence intervals (shown by colored stripes). Surprisingly, **Figure 5** shows a time trend that is the opposite of what we have seen in the frequency analysis (**Figure 3**). There is no difference between the groups in the first hour; so, the confidence intervals are overlapping completely. In the next 2 h, the bout durations decrease in each group; however, the decrease is much faster in the 10-mg/kg dose group than in the other two, particularly between the first and second hours. At the end of the observation period, the confidence intervals are clearly separated in left the panel (PW) of **Figure 5** but not in the right panel (S1), though the trend is similar in both panels. Extending the model with a time × treatment interaction term, this interaction was significant only for PW, and only in the 10-mg/kg dose group (F = 5.07, DF = 4, 1,350, p < 0.001).

### Sleep Microarchitecture Analysis

**Figure 6:**
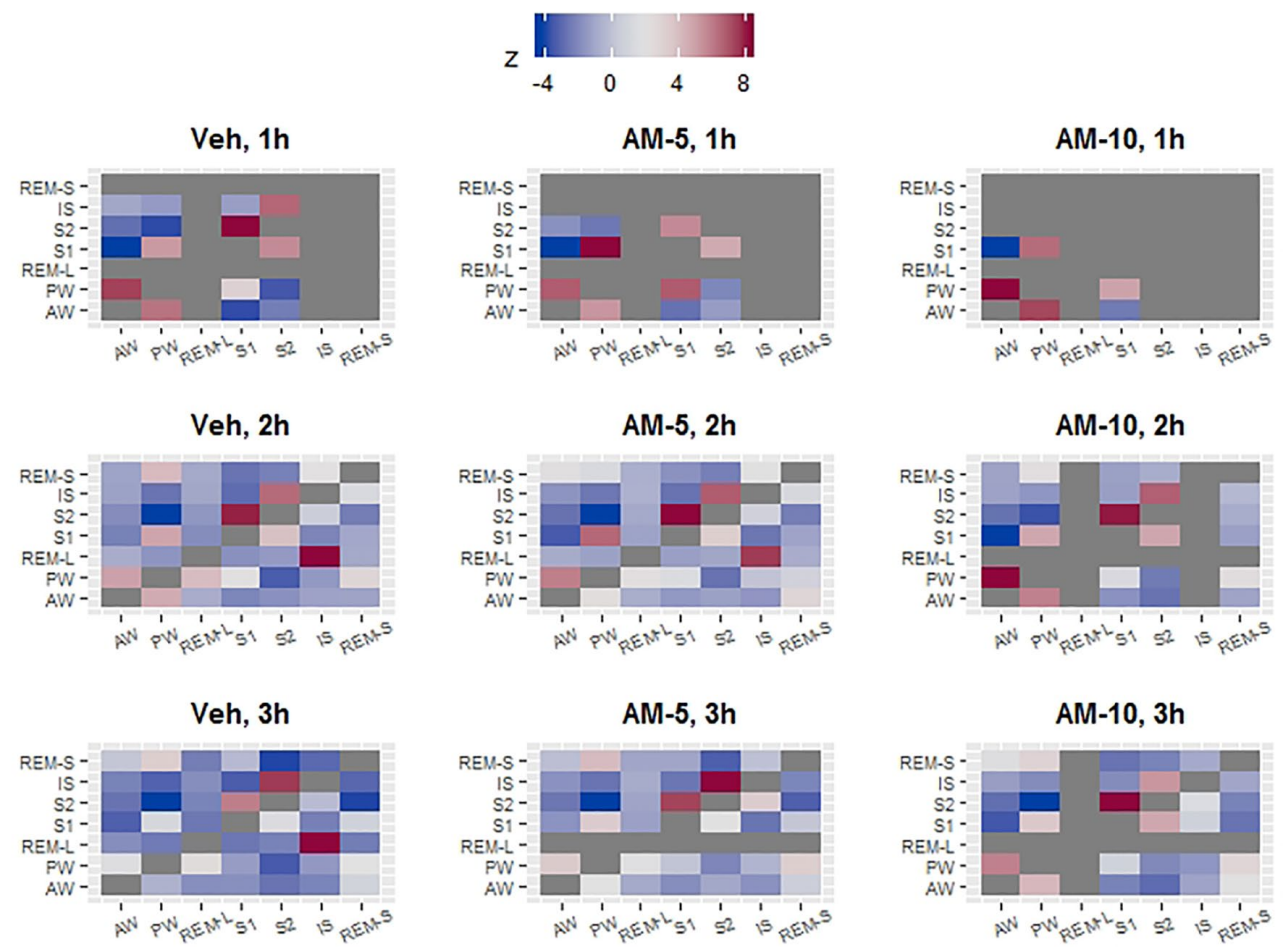
The vigilance states do not randomly follow each other. Deviation from random order was quantified with z statistics. A high positive z value (dark red) indicates that a given state on the Y axis is more likely to transit to another state (on the X axis) compared to all others. The dark blue fields represent the opposite, i.e., the given state (on Y axis) is very unlikely to transit to a stage (on the X axis). For example, if PW was observed in the vehicle group, then, the most likely next stages are AW or S1 but S2 is very unlikely. The z statistic is not estimable if there is no transition (diagonal tiles) or there are too few (less than 5) observations in the cell. In these two cases, the tiles were colored dark gray. Z values that are larger than 2 or smaller than -2 are usually considered significant; however, this rule is based on large sample approximation.

Both the frequency and duration of bouts conditionally depend on the characteristics of the previous bout (McShane et al., 2010; Bizzotto and Zamuner, 2018; Stephenson et al., 2013). For example, durations of REMS bouts depend on whether the previous state is wake or NREMS (McShane et al., 2010). Also, the probability that the next bout will be an REMS bout depends on whether the current state is wake or NREMS (McShane et al., 2013). Statistically speaking, the bout characteristics conditionally depend on the type of the preceding bout. **Figures 3** and **4** demonstrate that AM-251 modulates bout frequencies rather than bout durations; however, this shows only an overall picture, by averaging the conditional effect. **Figures 3** and **4** do not provide information about the frequency of the state transitions that conditionally depends on the previous state. This statistical problem can be reformulated using pharmacological terminology: given that the animal is now in PW, how does AM-251 influence the chance whether the next stage would be AW and not S1? Or generally, how does the treatment divert the normal transition pathways? To answer these questions, first, we cross-tabulated the bout frequencies for every hour of the three-hour observation period and for each treatment. As a result, we got nine contingency tables which contained zeros in the diagonals due the variable definitions. In the next step, similarly to Kishi et al. (2008), we calculated the observed transition probabilities by dividing the table entries with the total number of state transitions. The observed transition probabilities can be directly interpreted. They show the probability of current and the next vigilance stages (i and j, respectively). If the transition process is completely random, the transition probabilities in row i and column j (p_ij_) are the product of the column and row marginals (p_.j_ and p_i_). The difference between the observed and the “no preference” predicted probabilities measures the specificity of i→j transition, i.e., how the transition from state i to state j is preferred compared to all other “j”-s. However, this difference still needs to be normalized, because a probability difference 0.05 can be very impressive if p_ij_ = 0.1 and irrelevant if p_ij_ = 0.85. The normalized difference, the so-called z-statistic, is widely used in categorical data analysis to measure associations between variables. In the context of this study, minus z means that the i -> j transition is less likely to happen compared to the completely random alternative, meaning that the transition is somehow inhibited. Conversely, a high positive z is a sign of a preferred pathway. In **Figure 6**, the preferred and inhibited transitions between states are visualized by mapping z values to color a scale. The gray color in **Figure 6** corresponds to those two cases when the z-s are not estimable because of the definition of variables (these are diagonal elements) or because there are fewer than five observations per cell. With the help of **Figure 6**, we can derive the probable bout trajectories by going row-wise and column-wise. For example, we can start in row-wise direction from PW. In the control group, in the first hour (upper left heat map), most of the traffic goes to AW (first column, dark red) followed by S1. The transition to S2 is strongly inhibited (dark blue). From S1, we can go back to PW (rose) or go further to S2 (also rose). Using the same upper left heatmap, we can also go in column-wise from bottom to up. Starting again from PW, we now see that PW is initiated mostly by impulses from AW (dark rose) followed by S1. S2 is dark blue again, suggesting that PW practically neither sends nor receives impulse from the state S2 directly. During the 3-h observation period, the traffic between AW and PW gradually redirected from dark red to close to neutral (white) in the Veh group, while it remains strongly connected in the treated groups. At the 5 mg/kg-dose level, the most prominent effect in the first hour is the lack of IS bouts followed by the disappearance of S2 bouts after the larger dose. **Figure 6** reveals several features that justify the distinction between REMS-S and REMS-L. REMS-L is very likely followed by an IS bout, which is not true for REMS-S. Both REMS-S and REMS-L bouts are suppressed in the first hour in all treatment groups, but the suppression remains prolonged only for REMS-L.

## Discussion

Santucci et al. (1996) have demonstrated that the CB1 antagonist SR141716A dose-dependently increased the time spent awake with a parallel suppression of NREMS and REMS in rats. Goonawardena et al. (2011) could not confirm these findings in rats with applying AM-251 in 2-mg/kg dose, although they have observed an increased REMS latency that suggests an inhibition of REMS generation. However, testing AM-251 and ABD459 (another CB1 antagonist) in 3-mg/kg dose showed that both drugs produced REMS-decreasing effect in mice, while wakefulness was increased by AM-251 only (Goonawardena et al., 2015). In a recent study, dronabinol, a nonselective agonist of CB1/CB2 receptors, has been shown to decrease the percent time of REMS, and this effect was not reversible by either AM-251 or the CB2 receptor antagonist AM-630. Also, none of the CB1 and CB2 receptor antagonists could modify the sleep–wake pattern and the bout durations of NREMS and REMS items by applying alone (Calik and Carley, 2017). All these studies drew their conclusion by classifying vigilance states to REMS, NREMS, and wakefulness. For statistical comparisons, the authors used basic statistical tools, which, in principle, are applicable only for continuous variables (Santucci et al., 1996; Goonawardena et al., 2011; Goonawardena et al., 2015; Calik and Carley, 2017). This is the so-called standard or conventional approach (McShane et al., 2010), which is widely criticized for not being sensitive enough and therefore leading to information loss. Several authors (Bizzotto et al., 2010; Kostyalik et al., 2014, Diniz Behn et al., 2010, Stephenson et al., 2013) suggested sophisticated models to overcome this problem. However, the proposed models require estimating a number of parameters, which is not an easy mathematical problem. The mathematical difficulties are multiplying in the presence of a drug effect that introduces additional time-dependent variations. This explains why these sophisticated models are very rarely used to assess drug effects. However, the simple approach proposed by McShane et al. (2010) seemed to provide a bypass from this deadlock, so we decided to test it using our data obtained with AM-251. First, we confirmed, in line with the literature, that AM-251 dose-dependently increases the time spent in AW at the expense of the amount of other states (**Figure 1**). **Figure 1** represents our results obtained by the standard approach, and we started our exploratory investigations with plotting the histograms of bout durations (**Figure 2**). **Figure 2** confirms that the distribution of bout durations in rats shows the same “spike and slab” characteristics described originally by McShane et al. (2010) in mice. This mixture distribution feature was the most pronounced for REMS, so we relabeled the REMS episodes as short and long REMS (REMS-S and REMS-L, respectively) using 16 s (four epochs) as a cutoff. Note that the standard practice is to exclude all short REMS data from the analysis (Kitka et al., 2009). **Figure 2** gives the impression that the shape of bout distributions in different vigilance stages remains unaffected by the treatments. If AM-251 had any effect on the average length of bout episodes, then, this effect would be apparent in **Figure 2** by observing that the histograms of the treated groups are shifted horizontally relative to the control. However, such an effect could not be observed, which suggests that the frequencies rather than the duration times of bouts were affected. Therefore, in the next step, we focused on the relationships between time, drug treatment, and the frequencies of state transitions. **Figure 3** shows the observed number of bouts by time and treatments. Strong time and dose dependency can be observed, practically for all vigilance states except PW. It is also noteworthy that, particularly in the first hour, several vigilance states, such as REMS-S, REMS-L, S2, and IS, are completely suppressed by the treatments. The ratio of bout numbers compared to the vehicle group is a plausible measure to assess the drug effect. We estimated this parameter using negative binomial regression (**Figure 4**). **Figure 4** shows that the smaller dose of AM-251 selectively suppressed the REMS-L bouts, while increased the AW frequency moderately. With the double dose, the frequency of AW bouts is roughly doubled, while the frequency of all other states drops by 25–60% except PW. The effect of the drug on REMS-L is not significant, because the confidence interval width is too wide. The probable reason of the wide confidence interval for REMS-L is that both REMS-S and REMS-L bouts are suppressed even in the vehicle-treated group, due to circadian factors in the regulation of the normal sleep–wake pattern. For this reason, the first hour of the study is uninformative (there is nothing to suppress), and there are too few observations. The analysis of the bout frequencies is based on the assumption that the multiplying effect of AM-251 on bout frequencies does not change with time. This is a reasonable assumption but could not be verified, because the regression procedure did not converge when the time × treatment interaction was included into the model. The probable reason of this, as shown in **Figure 6**, is that, particularly in the first hour, some type of bout events never happened or happened only for a very few times. For these vigilance states, the estimates are on the boundaries of the allowable parameter space and the optimization procedure is deemed to fail (Williamson et al., 2013). Retrospectively, setting the time of drug administration at periods when the REMS activity is high would have been better to estimate the drug effect on the REMS periods. Indeed, we followed vigilance states only for 3 h after drug administration, which seems to be a limitation of our study compared to Santucci et al. (1996) and Goonawardena et al. (2015) who applied longer periods, 4 and 6 h, respectively. However, we used AM-251 only as a tool to study the sleep effects of CB1 blockade effect. From this perspective, the change in concentration from pharmacokinetic reasons is a disturbing factor which interferes with the correct the evaluation of data. The estimated half-life of AM-251 is quite long (22 h) in rats (McLaughlin et al., 2013). Assuming a simple one-compartment model, this value gives that the relative difference between the initial maximum and the minimal concentration at the end of the observation period is 9.01%. Therefore, it is reasonable assuming that during the three-hour observation period, the plasma levels were relatively constant, and the time-dependent effects seen in **Figures 3**, **5**, and **6** are not the result of the drug elimination.

The results presented so far suggested that the effect of AM-251 only modulates the between-states transitions. To check the lack of effect on bout duration, a regression analysis was conducted between bout duration and by treatment for each vigilance state. The effect of AM-251 on the bout duration of PW was highly significant, which is surprising, as PW is the only vigilance state that was completely resistant to the effect AM-251 so far. However, we do not believe that this is a direct effect of AM-251 on neural circuits that are responsible for PW. We think this effect is a compensatory response as the difference from the control increases with time (**Figure 5**). If this were a direct dose-related effect, then the time course would be the opposite. Essentially, the same effect was observed for S1. However, in this case, the compensatory decrease in bout duration was much more moderate (**Figure 5**).

McShane et al. (2013) introduced and demonstrated the usefulness of conditional analysis that is computing bout characteristics conditionally on the previous state. Since we have already known that AM-251 affects only the transition frequencies but not the state lengths, we investigated only the conditional dependence of transition frequencies. In the first step of the conditional analysis, we made a 7 × 7 table where the number in row i and column j showed the bout number of state j when the predecessor was state i. The “7” comes from the fact that we distinguished seven vigilance states, and states i and j can be any of them. This kind of table is known as a contingence table in statistics, and many methods were developed to visualize it. The goal of the visualization is to find connection between categorical variables which translates them, in the current context as that state i and state j (variables) are connected if state i precedes state j. We measured the strength of the connection with the help of z-statistics. High z_ij_ statistics means that state_j_ is preferred over other states to “jump to” from the current state “i.” Large negative z means just the opposite—namely, the transition from “i” to “j” is not likely. When the matrix of z-s was visualized by mapping the z_ij_ -s to color, the resulting “transition heatmaps” turned to be very informative (**Figure 6**). Most of the z values were significantly positive and significantly negative (above 2 or below -2, respectively). Therefore, transitions between states generally cannot be considered random. In fact, we identified several regular features, characteristics for each map regardless of time or drug treatment. The heatmaps helped us easily discover highly connected vigilance stages and derive the likely bout trajectories. The drug, time, and circadian effect can be easily spotted on them. Taken together, we recommend using the transition heatmaps as explorative aids for the visualization of transitions between vigilance stages that can be a useful in the microstructure analysis of sleep research.

## Conclusion

AM-251 selectively modulates the frequency of transitions between vigilance stages without affecting the bout durations. It increases the transition to AW and suppresses the transitions to all other stages except to PW. Conversely, the durations of PW episodes are decreased, but the profile of this effect suggests an indirect compensatory action. The effect on sleep transitions was visualized using a newly developed tool we call transition heatmap. We believe that the easily computable transition heatmaps can be useful for exploring the microstructure of sleep–wake architecture.

## Data Availability

The datasets generated and/or analyzed during the current study are not publicly available due to ongoing analysis for future publication, but are available from the corresponding author upon reasonable request.

## Ethics Statement

All animal experiments and housing conditions were carried out in accordance with the EU Directive 2010/63/EU and the National Institutes of Health “Principles of Laboratory Animal Care” (NIH Publications No. 85-23, revised 1985), as well as specific national laws (the Hungarian Governmental Regulations on animal studies 40/2013). The experiments were approved by the National Scientific Ethical Committee on Animal Experimentation and permitted by the government (Food Chain Safety and Animal Health Directorate of the Central Agricultural Office, Permit No. 22.1/1375/7/2010).

## Author Contributions

GB and SV performed the experimental design. EB, NP, and SV performed the experimental procedures. EB and LT were involved in the analysis of the data. SV and LT wrote the manuscript. All the authors participated in the critical revision.

## Funding

Support for this research was provided by the National Development Agency Hungarian Brain Research Program (Grant No. KTIA_13_NAP-A-II/14), by NAP 2.0 (Grant No. 2017-1.2.1-NKP-2017-00002) and by EFOP-3.6.3-VEKOP-16-2017-00009 grant.

## Conflict of Interest Statement

The authors declare that the research was conducted in the absence of any commercial or financial relationships that could be construed as a potential conflict of interest.
